# Preparation of medical hydrophilic and antibacterial silicone rubber *via* surface modification†

**DOI:** 10.1039/d1ra06260c

**Published:** 2021-12-15

**Authors:** Kaidi Ding, Yue Wang, Shuaizhen Liu, Sen Wang, Jianguo Mi

**Affiliations:** Beijing Laboratory of Biomedical Materials, Beijing University of Chemical Technology No. 15 Beisanhuandong Road Beijing 100029 China mijg@mail.buct.edu.cn +86-010-64434788; State Key Laboratory of Organic-Inorganic Composites, Beijing University of Chemical Technology No. 15 Beisanhuandong Road Beijing 100029 China

## Abstract

Bacterial adhesion of medical indwelling devices shortens their service life and brings about infections in patients. The combination of hydrophilic and antibacterial modifications can not only kill the bacteria in contact with the surface, but also avoid the adhesion of dead bacteria. From this view, with a self-made quaternary ammonium salt and a zwitterion as the modifiers, a modified silicone rubber, SR-*g*-(DMAPS-*co*-QA), was prepared *via* random co-grafting. The static water angle test and antibacterial assays proved the enhancement of both the hydrophilicity and antibacterial activity. In addition, compared with the unmodified silicone rubber, after 7 days of co-cultivation in *E. coli* suspension, SR-*g*-(DMAPS-*co*-QA) demonstrated good resistance to biofilm formation. Furthermore, to simulate the real situation, its antibacterial effect in dynamic flow condition was measured, confirming that SR-*g*-(DMAPS-*co*-QA) still maintained good antibacterial performance after a 48 hour cyclic flow of *E. coli* bacterial suspension.

## Abstract 2

Surface modification of silicone rubber to enhance hydrophilicity and antibacterial effect.

## Graphical abstract

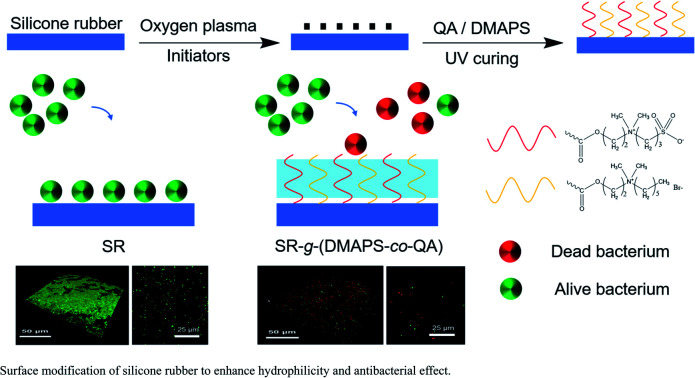

## 1. Introduction

Silicone rubber is a polymer whose main chain is composed of silicon and oxygen atoms.^1^ It demonstrates good biocompatibility without any toxicity or odor.^2^ In addition, as the bond energy of Si–O is higher than that of both C–O and C–C,^3^ silicone rubber is recognized as an ideal material in medical and clinical areas, such as indwelling devices and implants.^4–6^

When a medical device is implanted in a human body, the existence of bacteria will not only lead to deterioration of the material,^7^ but also induce serious infections.^6–10^ In the view of microbiology, the keys to inhibit bacteria on the surface of a medical device are to alleviate the adhesion of bacteria effectively and hinder the formation of biofilms, or to kill the bacteria in contact with the surfaces.^11–13^ An effective approach is to construct hydrophilic and antibacterial surfaces *via* modification.

A hydrophilic substance demonstrates high surface tension with water at the interface, thus could attract water molecules strongly and form a protective hydration layer, which could prevent the adhesion of microorganisms.^14^ In addition, the hydrophilic surface could also decrease the adhesion between the implanted device and the tissues in the body, thereby relieve the pain of the patient.^15^ Considering the high hydrophobicity of silicone rubber, the hydrophilic modification is of great necessity.^4,16^ One of the common techniques is surface grafting.^17–19^ The existence of chemical bonds between the substrate and the hydrophilic substances could equip the surface with stable and long-lasting hydrophilicity. Among various hydrophilic modifiers, zwitterions, molecules containing both cations and anions, could bind water molecules through electrostatic interactions tightly and induce hydration, and demonstrate prefect resistance to nonspecific protein adsorption.^20–22^ Currently, zwitterions are widely used in areas with high demand of antifouling performance, such as sewage treatment and seawater desalination.^20–24^ Although current zwitterionic approaches demonstrate great potentials and attract worldwide attentions, few literatures reported the industrialization of this surface modification method.

To solve the bacterial infections caused by indwelling and implanted devices, the antibacterial functionalization of materials is imperative in clinical areas.^25–28^ In comparison with physical modification, chemical modification is not restricted by the compatibility between the continuous and the dispersed phases, and antibacterial modifiers hardly leach out from the matrix.^29^ For the selection of the antibacterial agents, quaternary ammonium salts have broad-spectrum antibacterial effect and high antibacterial efficiency, which make it applicable in medical, food packaging, coatings and other fields.^29–31^

Hydrophilic modification effectively reduces the adhesion of microorganisms at the interface, but it cannot kill the bacteria that have adhered to the surface, which will steadily aggregate, propagate and eventually form a biofilm. On the other hand, the antibacterial modification immediately eliminates the bacteria in contact with the surface, but the dead bacteria colonies and their intracellular substances flowing out after death will stack and adhere to the surface, leading to a weakening effect of the antibacterial property.^32,33^ From this perspective, this paper focused on the preparation of a modified silicone rubber with both high hydrophilicity and good antibacterial performance. A self-prepared quaternary ammonium salt (QA) and a zwitterion, [2-(methacryloyloxy)ethyl]dimethyl-(3-sulfopropyl)ammonium hydroxide (DMAPS) were selected as the antibacterial modifier and hydrophilic modifier for the surface grafting, respectively. The hydrophilicity and antibacterial performance of the modified silicone rubber were characterized. Furthermore, considering the potential applications of this modified silicone rubber, the antibacterial performance under a dynamic flow condition were measured for and overall evaluation.

## 2. Materials and methods

### 2.1. Materials

Vinyl methyl silicones (VMQ, SR-35, medical grade), hydrogen-containing silicon oil (medical grade), and vulcanizer (type A, medical grade) were obtained from Tuoren Medical Device Co., Ltd, Henan, China. 1-Bromohexane (96%), 2-(dimethylamino)ethyl methacrylate (DMAEMA, 99%) and [2-(methacryloyloxy)ethyl]dimethyl-(3-sulfopropyl)ammonium hydroxide (DMAPS, 99.5%) were purchased from Energy Chemical, Shanghai, China. Benzophenone (BP, 99%) was purchased from Zhanyun Chemical Co., Ltd, Shanghai, China. 2,2-Dimethoxy-2-phenylacetophenone (DMPA, 99%) and acetonitrile (94%) were purchased from Aladdin Biochemical Technology Co., Ltd, Shanghai, China. Isopropyl ether (AR) and ethanol (AR) were purchased from Beijing Institute of Chemical reagents, Beijing, China. *Escherichia coli* (*E. coli*, ATCC 25922) and *Staphylococcus aureus* (*S. aureus*, ATCC 25923) were used for antibacterial assays.

### 2.2. Preparation of vulcanized VMQ (SR)

VMQ was fed into a two-roll mill (XH-401B, Dongguan Xihua Testing Machine Co., Ltd, Guangdong, China) at room temperature. After 1 min, hydrogen-containing silicone oil was added into the mill thrice, with amounts of 0.6, 0.8, and 1.0 wt% of the VQM successively. Then after 5 min, the vulcanizer with an amount of 0.6 wt% of the VQM were added. After another 3 min, the nip gap was adjusted, and sheets of VQM blend was obtained. The vulcanization was operated in a plate vulcanizer (XH-406, Dongguan Xihua Testing Machine Co., Ltd, Guangdong, China) with a temperate of 110 °C for 4.5 min. After cooling down, SR sheets with a thickness of 1 mm were obtained.

### 2.3. Synthesis of QA

10.75 mL of 1-bromohexane and 10.7 mL of DMAEMA were added into 40 mL of acetonitrile in a single-neck flask and reacted at 40 °C under magnetic stir with a speed of 350 rpm. After 24 h, the solution was transfer into a rotary evaporator. The solvent was evaporated until the remaining solution was about 20 mL. After the residue concentrate was washed with about 200 mL isopropyl ether and centrifuged with a rotation speed of 3300 rpm for 3 min, the precipitate was reserved. This procedure was repeated thrice. The precipitate was dried in a vacuum oven at room temperature for 24 h, and then, the white powder, the quaternary ammonium salt (QA), was obtained.

### 2.4. Surface modifications of SR

To achieve the best balance between hydrophilicity and transparency of the modified SR, BP and DMPA were used as the photo-initiators, and the UV curing period was 3 min. Detailed information could be found in SI. BP and DMPA were dissolved in acetone with amounts of 4.5 and 0.5 wt% of the acetone, respectively. The SR sheets were cut into square films with a size of 2 × 2 cm, and washed in 50% ethanol solution in an ultrasonic cleaner (KQ5200DE, Kunshan Ultrasonic Instrument Co. Ltd, Jiangsu, China) for 10 min. After dried at atmosphere and treated with oxygen plasma for 5 min, the films were then immersed into the BP/DMPA/acetone solution, and sealed in a water bath at 37 °C. After 2 h, the films were taken out and dried at atmosphere for 1 h.

Three water solutions were prepared: the DMAPS solution with a concentration of 0.3 g mL^−1^, the QA solutions 0.6 g mL^−1^, and the DMAPS/QA solution with a DMAPS concentration of 0.3 g mL^−1^ and a QA concentration of 0.6 g mL^−1^. The treated SR films were placed into the different solutions and cured in a UV curing equipment (KW1.5, Chemat Technology Inc., Shanghai, China) separately with a UV wavelength of 365 nm and an energy density of 6 mW cm^−2^. After 3 min, the cured SR films were ultrasonicated for 1 min to remove the unreacted monomers and initiators. Then, the modified SR, SR-*g*-DMAPS, SR-*g*-QA and SR-*g*-(DMAPS-*co*-QA), were obtained.

### 2.5. Structural characterizations

The chemical structure of QA was confirmed *via* a ^1^H nuclear magnetic resonance (NMR, AVANCE III, Bruker Corp., MA, USA). The test was conducted at room temperature with D_2_O as the solvent. The chemical structures of SR, SR-*g*-DMAPS, SR-*g*-QA, and SR-*g*-(DMAPS-*co*-QA) were confirmed *via* an attenuated total reflectance-Fourier transform infrared (ATR-FTIR, Nicolet iS5, Thermo Fisher Scientific, MA, USA). The test was performed in a scan range of 500–4000 cm^−1^. The surface chemical structures of SR, SR-*g*-DMAPS, SR-*g*-QA, and SR-*g*-(DMAPS-*co*-QA) were determined through the elemental analysis using an X-ray photoelectron spectroscopy (XPS, ESCALAB 250, Thermo Fisher Scientific, MA, USA). Al Kα X-ray was used as the radiation source, the pass energy was set as 20 eV, and the take-off angle was set as 90°. The XPS data were analyzed using Avantage.

### 2.6. Statistic water contact angle experiment

Before the test, the surfaces of the SR, SR-*g*-DMAPS, SR-*g*-QA, and SR-*g*-(DMAPS-*co*-QA) films were swiped with a 75% ethanol solution and placed on clean glass slides. The test was operated in a contact angle meter (OCA50Micro, Jinhe Instrument Co., Ltd, Hebei, China). The water drop size was set as 0.3 μL. Five parallel samples were required for each test.

### 2.7. Antibacterial assays

Before all the antibacterial assays, all the containers should be disinfected in an autoclave steam sterilizer at 120 °C for 20 min, and the specimens should be sterilized in a UV disinfection cabinet for 10 min. The preparation of the culture solution and the solid culture medium conformed to Chinese standard QB/T 31402-2015.^34^ At least three parallel samples were required for each test. The bacteria were cultivated in the culture solution at 37 °C for 8 h until required concentrations were reached in advance.

#### 2.7.1. Direct contact experiment

32 μL of bacterial suspension with a concentration of 5 × 10^5^ cfu mL^−1^ was inoculated onto the surface of each SR, SR-*g*-DMAPS, SR-*g*-QA, and SR-*g*-(DMAPS-*co*-QA) film. Then the films were placed in an incubator at 37 °C. After 24 h, the bacterial suspension on the surface of each film was sucked, diluted and coated on the solid culture medium in a Petri dish. After incubation for another 24 h, the viabilities of the bacteria in the Petri dishes were observed. Detailed operations can be found in the ref. 35.

#### 2.7.2. Co-cultivation experiment

The SR, SR-*g*-DMAPS, SR-*g*-QA, and SR-*g*-(DMAPS-*co*-QA) films were cut into 4 × 4 mm specimens. Each specimen and the bacterial suspension with a concentration of 10^7^ cfu mL^−1^ were placed into a sealed sterilized container and co-cultivated in an incubator shaker at 37 °C. After 12 h, the bacterial suspensions were sucked and stained with SYTO9 and PI respectively and observed in a confocal laser scanning microscopy (CLSM, Leica, Leica Camera AG, Wetzlar, Germany). Detailed operations can be found in the ref. 35.

### 2.8. Biofilm formation experiment

The SR, SR-*g*-DMAPS, SR-*g*-QA, and SR-*g*-(DMAPS-*co*-QA) films were cut into 4 × 4 mm square specimens. Each specimen and 200 μL of *E. coli* suspension with a concentration of 10^8^ cfu mL^−1^ were placed into a sealed sterilized container and co-cultivated in an incubator shaker at 37 °C. After 7 days, the bacterial suspensions were sucked and stained with SYTO9 and PI respectively, and observed using a CLSM.

### 2.9. Antibacterial activity of SR-*g*-(DMAPS-*co*-QA) under the flow condition

Approximately 150 mL of *E. coli* suspension with a concentration of 10^7^ cfu mL^−1^ was added into a beaker. A catheter passed across a peristaltic pump, with its the two ends immersing in the suspension. The SR and SR-*g*-(DMAPS-*co*-QA) films were cut into specimens with appropriate sizes, so that they just fit the shape of the catheter and were placed inside it without any blockage. With the adjustment of the peristaltic pump, the suspension was cyclically flowed through the catheter with a flow rate of 5 mL min^−1^ at 37 °C. The circulating *E. coli* suspension in the beaker was replaced by a fresh one in every 24 hours. After 48 hours, the *E. coli* suspensions were stained with SYTO9 and PI respectively, and observed using a CLSM.

## 3. Results and discussion

**Fig. 1:**
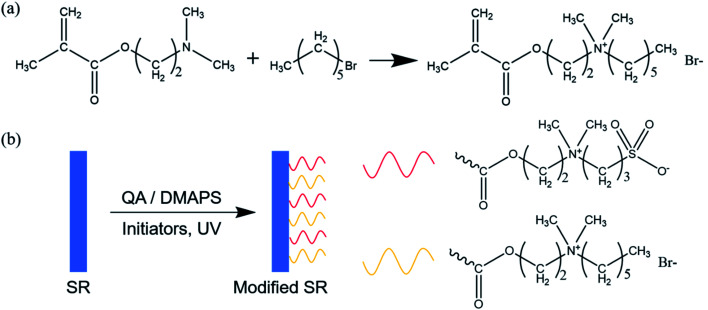
Schematic of preparation of hydrophilic antibacterial SR: (a) synthesis of QA, (b) surface grafting of SR with QA and DMAPS.

The experimental flow chart is shown in Fig. 1. QA was synthesized *via* the quaternization reaction between 1-bromohexane and DMAEMA, as is shown in Fig. 1(a). In the surface modification, under UV light, initiated by BP and DMPA, the surfaces of the SR were activated, thus large amounts of radicals existed. The plasma treatment in advance could increase the interactions between the initiators and the surfaces. With the help of the radicals, QA and DMAEMA were grafted onto the surfaces randomly, as is shown in Fig. 1(b).

### 3.1. Structural analysis

**Fig. 2:**
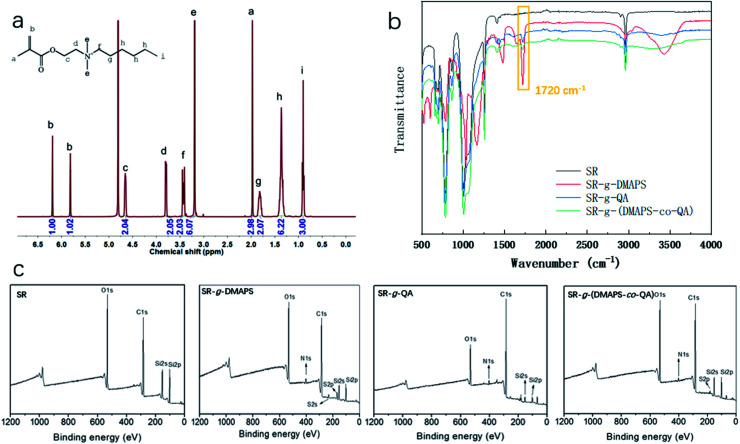
(a) ^1^H NMR spectrum of QA, (b) FTIR and (c) XPS spectra of SR, SR-*g*-DMAPS, SR-*g*-QA, and SR-*g*-(DMAPS-*co*-QA).

Fig. 2(a) is the ^1^H NMR spectrum of QA. The adsorption peaks in the range of 3.2 to 3.8 ppm are ascribed to the protons in the methyl and methylene groups adjacent to N^+^, so that are the characteristic peaks of quaternary ammonium salts.^36^ According to the corresponding relationships between the chemical groups and the peaks, the area ratio of different peaks, which represents the ratio of protons in different groups, is approximately in accordance with the chemical structure of QA. The result proved the successful synthesis of QA.

To verify whether DMAPS and QA were grafted onto SR, FTIR and XPS were conducted, and the results are shown in Fig. 2(b) and (c). In the FTIR spectra, new peaks at approximately 1720 cm^−1^ appeared in the spectra of modified SR. This peak represents the stretching vibrations of carbonyl groups. According to the schematic in Fig. 1(b), this result indicates the successful graft of SR. Furthermore, in the XPS spectra of the modified SR, peaks representing N 1s and S 2p appeared, and the signal of the peaks representing Si 2s decreased significantly, proving the introduction of DMAPS and QA on the surfaces of SR.

### 3.2. Static water contact angle analysis

**Fig. 3:**
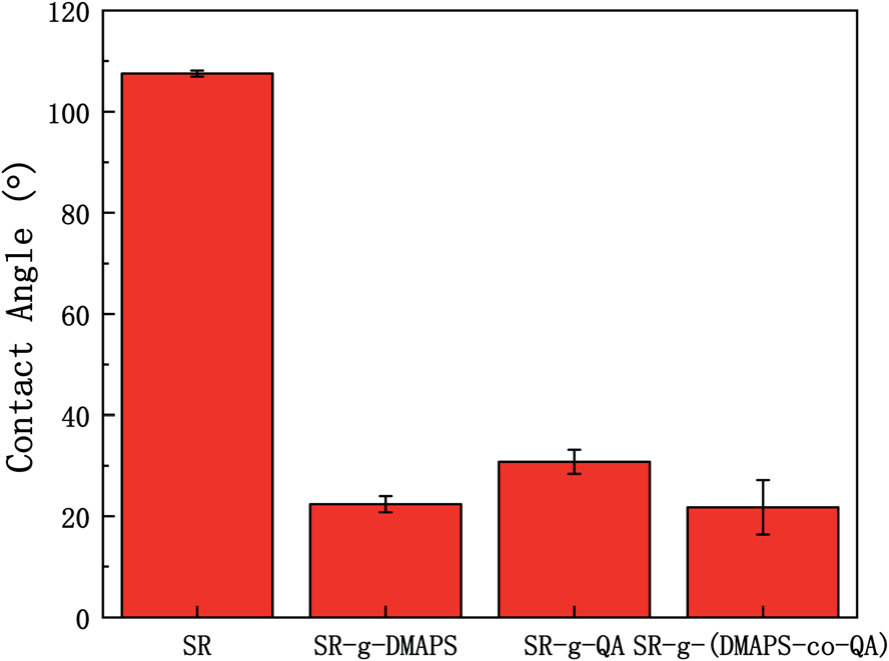
Static water contact angle of SR, SR-*g*-DMAPS, SR-*g*-QA, and SR-*g*-(DMAPS-*co*-QA).

The static water contact angles of SR, SR-*g*-DMAPS, SR-*g*-QA, and SR-*g*-(DMAPS-*co*-QA) are illustrated in Fig. 3. SR was hydrophobic with a water contact angle of 107.5°. After modification, the water contact angle decreased significantly, indicating a great improvement of hydrophilicity. This phenomenon is attributed to the sulfonic acid group in DMAPS and the quaternary ammonium group in QA. The sulfonic acid group has a stronger hydrophilic effect than that of the quaternary ammonium group, leading to a lower water contact angle of SR-*g*-DMAPS. On the surface of SR-*g*-(PDMAS-*co*-QA), with the joint effect of PDMAS and QA, the water contact angle decreased to about 21.8°, indicating that the hydrophilicity was significantly enhanced.

### 3.3. Antibacterial activity analysis

**Fig. 4:**
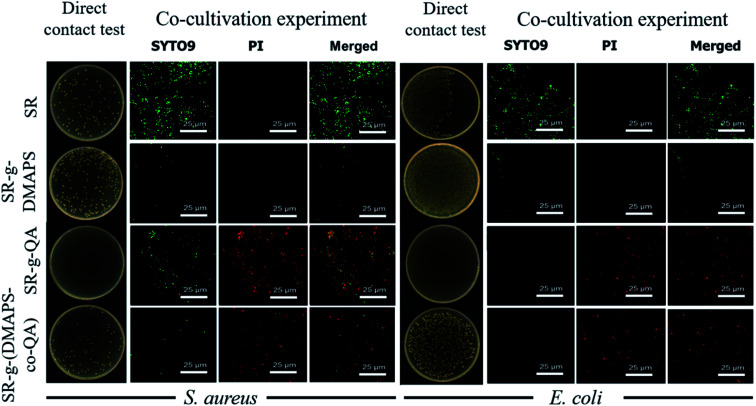
Antibacterial effects of SR, SR-*g*-DMAPS, SR-*g*-QA, and SR-*g*-(DMAPS-*co*-QA) in direct contact and co-cultivation experiments.

As is shown in Fig. 4, two methods were used to characterize the antibacterial performance of the SR, SR-*g*-DMAPS, SR-*g*-QA, and SR-*g*-(DMAPS-*co*-QA): direct contact and cocultivation experiments. In the direct contact experiment, SR-*g*-QA demonstrated the best antibacterial performance against both *S. aureus* and *E. coli*, which is attributed to the existence of quaternary ammonium groups, while SR-*g*-DMAPS showed no obvious antibacterial activity, suggesting no antibacterial effect of DMAPS. Although SR-*g*-(DMAPS-*co*-QA) still could resist the growth and propagation of bacteria, its antibacterial property was weaker than that of SR-*g*-QA, because DMAPS some occupied active cites and radicals, leading to a decreased grafting ratio of QA. In the CLSM images in co-cultivation experiment, the result was similar, except that the amount of the bacteria colonies, which were all alive, was comparably small on the surface of SR-*g*-DMAPS in the view. This is attributed to its high hydrophilicity, so that few bacteria could adhere on the surface.^14^ In contrast, large amount of alive bacteria colonies was found on the other surfaces. It should be noted that the antibacterial effects of the QA-grafted SR against *S. aureus* were much higher than that against *E. coli*. The antibacterial mechanism of quaternary ammonium salts is contact killing, thereby the cell wall structures make much difference to the antibacterial activities.^29^*S. aureus* is categorized as Gram-positive bacterium, while *E. coli* Gram-negative bacterium. As quaternary ammonium salts have higher resistance against Gram-positive bacterium,^37^ QA-grafted SR demonstrated higher antibacterial properties against *S. aureus*.

### 3.4. Biofilm formation analysis

**Fig. 5:**
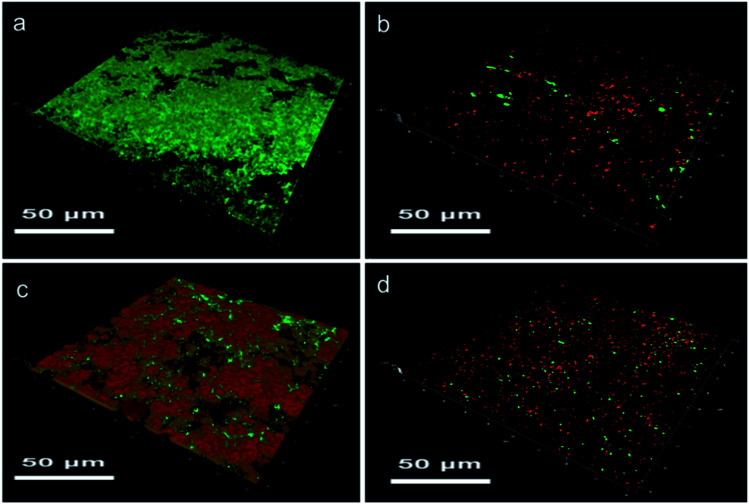
Biofilms on the surfaces of (a) SR, (b) SR-*g*-DMAPS, (c) SR-*g*-QA, and (d) SR-*g*-(DMAPS-*co*-QA) after co-cultivation with *E. coli*.

Biofilm formation experiment demonstrates the bacterial adhesion on a surface directly, so the results of SR, SR-*g*-DMAPS, SR-*g*-QA, and SR-*g*-(DMAPS-*co*-QA) are shown in Fig. 5. Bacteria were prone to adhere to the hydrophobic surfaces of SR, so severe biofilm of alive bacteria was found in the CLSM image after co-cultivation for 7 days. On the contrary, SR-*g*-DMAPS with high hydrophilicity could perfectly resist the adhesion of bacteria, so that only a few bacteria colonies were observed, of which some were alive colonies with large population. However, although SR-*g*-QA also showed low water contact angle, which was almost equal to that of SR-*g*-DMAPS, obvious biofilm was still formed on its surface. This is because that the surfaces of bacteria are negative-charged in most cases, thus the positive-charged quaternary ammonium cations attract bacteria *via* electrostatic force strongly. Considering the powerful antibacterial activity of QA, the biofilm on the surface of SR-*g*-DMAPS consisted largely of dead bacteria. When DMAPS and QA were co-grafted onto SR, after co-cultivation, no obvious biofilm was formed, and only some bacteria adhered to its surface. QA occupied radicals and the grafting ratio of DMAPS decreased, the total amounts of *E. coli* colonies on the surface of SR-*g*-(DMAPS-*co*-QA) was larger than that of SR-*g*-DMAPS, while colonies with large population disappeared. The co-graft of DMAPS and QA is a compromise of the hydrophilicity and antibacterial effect. Although perfect resistance to bacteria adhesion of SR-*g*-DMAPS is sacrificed, the growth and propagation of bacteria on the surface was greatly inhibited.

### 3.5. Antibacterial activity of SR-*g*-(DMAPS-*co*-QA) under the flow condition

**Fig. 6:**
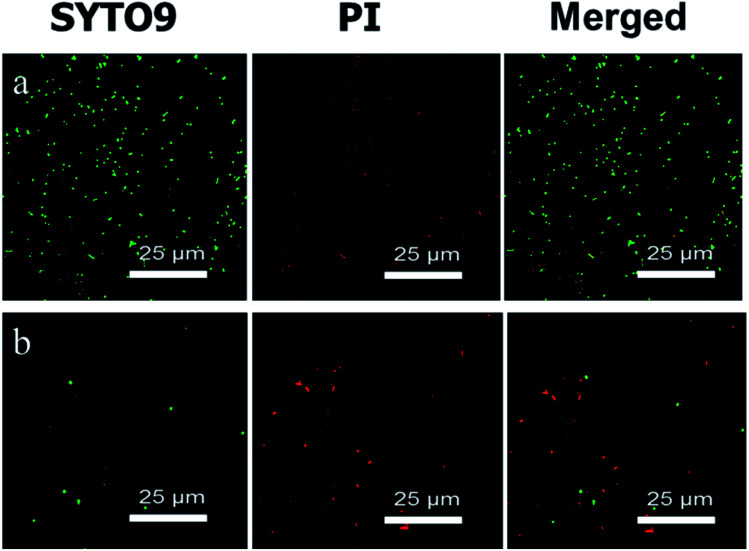
CLSM images of the surfaces of (a) SR and (b) SR-*g*-(DMAPS-*co*-QA) under flow condition.

Considering that the possible application of silicone rubber in medical areas, such as urinary catheters, tracheal intubation, intravenous catheters, *etc.*, an experimental device simulating the real circumstance was constructed, and the antibacterial effect of the SR-*g*-(DMAPS-*co*-QA) in dynamic flow condition was characterized, as is shown in Fig. 6. After 48 hours of cyclic flow, large amounts of alive bacteria adhered to the surface of SR, while only a few adhered to that of SR-*g*-(DMAPS-*co*-QA), of which mostly were dead. This result proves that SR-*g*-(DMAPS-*co*-QA) could prevent the adherence and growth of bacteria in the condition simulating cyclic flow in internal environment.

## 4. Conclusion

To achieve the improvement of both the hydrophilicity and antibacterial effect, this paper co-grafted QA and DMAPS onto the surface of the silicone rubber. The structures of the products were characterized by ^1^H NMR, FTIR and XPS, which proved that the preparation was successful, and both QA and DMAPS were introduced. After the modification, the static water contact angle of decreased from 107.5° to 21.8°, and the antibacterial performance was also elevated. After 7 days of co-cultivation with *E. coli*, only a few dead bacteria existed, and no obvious biofilm was formed on the surface of SR-*g*-(DMAPS-*co*-QA). In antibacterial assay simulating the real situation, SR-*g*-(DMAPS-*co*-QA) could resist the growth and propagation of *E. coli* under the dynamic flow condition within 48 hours. The modified silicone rubber demonstrated good antibacterial effect and resistance against bacterial adhesion, indicating a potential application in medical indwelling and implanting devices.

## Conflicts of interest

There are no conflicts of interest.

## Supplementary Material
